# Contrasting Social/Familial Networks with Caregiving Networks of Older Adults with Cognitive Impairment

**DOI:** 10.1093/geroni/igaf122.2648

**Published:** 2025-12-31

**Authors:** Amanda Leggett, Wenjing Li, Richard Gonzalez, Wenhua Lai, Natasha Nemmers

**Affiliations:** Wayne State University, Detroit, Michigan, United States; Wayne State University, Detroit, Michigan, United States; University of Michigan, Ann Arbor, Michigan, United States; Wayne State University, Detroit, Michigan, United States; Wayne State University, Detroit, Michigan, United States

## Abstract

A growing body of research is examining family caregivers’ social networks, yet how such networks translate into care provision is underexplored. This study extends the Task Specific Model of Caregiver Selection to 1) compare social/familial network membership with caregiver network membership and 2) explore whether demographics or care needs better predict the proportion of the network assisting with care. This study includes 742 community-dwelling individuals with cognitive impairment receiving care from the 2022 wave of the National Health and Aging Trends Study. Unpaired Wilcox tests compare social/familial network members with caregivers (e.g., number of children vs. number of children caregivers). Next, Poisson regressions predict the proportion of caregivers among one’s household, children, and social network members, with demographics (age, gender, race) and care needs (dementia status, functional disability, chronic medical conditions) as covariates. Significantly more household members, children, and social network members are in one’s network than are providing care. In regression models, being Black or Hispanic relative to White was associated with a smaller proportion of helpers in one’s household. Being older, female, Black (relative to White), and needing more transportation assistance was associated with a greater proportion of one’s children serving as helpers. Finally, needing more transportation and medical assistance was associated with a greater proportion of one’s social network assisting with care. Demographic characteristics of the individual with cognitive impairment related to greater proportions of family assistance, while greater care needs related to a greater proportion of one’s social network assisting with care.

